# Research progress of biomimetic materials in oral medicine

**DOI:** 10.1186/s13036-023-00382-4

**Published:** 2023-11-23

**Authors:** Xinyu Luo, Jiayue Niu, Guanyu Su, Linxi Zhou, Xue Zhang, Ying Liu, Qiang Wang, Ningning Sun

**Affiliations:** 1 Liaoning Provincial Key Laboratory of Oral Diseases, School and Hospital of Stomatology, China Medical University, No. 117 Nanjing North Street, Shenyang, 110001 China; 2 Department of Orthodontics, Shanghai Ninth People’s Hospital, Shanghai Jiao Tong University School of Medicine, Shanghai, China; 3 College of Stomatology, Shanghai Jiao Tong University, Shanghai, 200011 China; 4 National Center for Stomatology, Shanghai, 200011 China; 5 National Clinical Research Center for Oral Diseases, Shanghai, 200011 China; 6 Shanghai Key Laboratory of Stomatology, Shanghai, 200011 China

**Keywords:** Biomimetic materials, Biomineralization, Surface modification, Tissue engineering, Periodontal regeneration

## Abstract

Biomimetic materials are able to mimic the structure and functional properties of native tissues especially natural oral tissues. They have attracted growing attention for their potential to achieve configurable and functional reconstruction in oral medicine. Though tremendous progress has been made regarding biomimetic materials, significant challenges still remain in terms of controversy on the mechanism of tooth tissue regeneration, lack of options for manufacturing such materials and insufficiency of in vivo experimental tests in related fields. In this review, the biomimetic materials used in oral medicine are summarized systematically, including tooth defect, tooth loss, periodontal diseases and maxillofacial bone defect. Various theoretical foundations of biomimetic materials research are reviewed, introducing the current and pertinent results. The benefits and limitations of these materials are summed up at the same time. Finally, challenges and potential of this field are discussed. This review provides the framework and support for further research in addition to giving a generally novel and fundamental basis for the utilization of biomimetic materials in the future.

## Inroduction

Tooth defect, tooth loss, periodontal diseases, flaws in the maxillofacial bone tissue, and other conditions affecting the oral tissues continue to be prevalent [1–4]. Nowadays, various materials are employed in the treatment of these diseases. However, it is difficult to replicate the structural characteristics of native tissues because the physical and chemical properties of natural human tissues are greatly different from those of the materials [5]. Moreover, there are problems such as mismatched material performance, long degradation time, and immune rejection, which lead to unsatisfied clinical treatment effects. With the increasing demand for more innovative, stable, and reliable materials in the field of oral medicine, the application of biomimetic materials will provide new solutions and development directions for these clinical problems. Biomimetic materials, with the aim to reconstruct products as close as possible to natural human tissues, have emerged with the rapid development of biomimetic technology and continuous penetration in various research fields. The researches of biomimetic materials in the field of oral medicine focus on the development of materials with better mechanical properties and biocompatibility that mimic the characteristics of natural oral soft and hard tissues [6, 7].

The recent research progress and application of biomimetic materials are elaborated from four parts: the biomimetic materials for treating tooth defect, tooth loss, periodontal diseases and maxillofacial bone defect. For the part of tooth defect, enamel and dentin biomimetic remineralization materials as well as biomimetic restoration materials are mainly presented. Through imitating the crucial process of mineralization, the bottom-up induction of remineralization can achieve the reconstruction of enamel and dentin. Compared with conventional ones, biomimetic restoration materials have properties akin to those of tooth tissue. Dental Implantation, as currently the most common therapeutic approach for tooth loss, often leads to unsatisfactory treatment outcomes due to the risk of infection [8]. The biomimetic coatings and biomimetic nanostructures on the implant surface have positive effect to prevent infection as well as reduce the dependence on antibiotics [9]. Recently, numerous studies on materials related to tooth regeneration are great substitutes to traditional implants, which may provide a new method for tooth loss [10–12]. Given the loss of periodontal tissue due to periodontal disease, guided tissue regeneration (GTR) technology is widely performed in clinical application [13]. Unfortunately, the technology relies self-healing ability of tissue too much. This limitation provides the foundation for the development of biomimetic materials of periodontal tissue regeneration-an effective material for periodontal tissue regeneration that, crucially, promotes cell differentiation with its own properties. What's more, biomimetic bone substitute materials are ideally adapted to the maxillofacial bone defect. In contrast to other autologous or allogeneic bone, they reduce the problems related with high autologous bone absorption rates, donor site complications, and allogeneic bone immunological rejection [14].

Nowadays, the research and application of biomimetic materials are in the preliminary exploration stage, meaning that there are still many shortcomings that need to be improved [15]. This review summarizes the previous works in the field of biomimetic materials in oral medicine particularly nascent materials and corresponding manufacturing technologies. Finally, key issues in this area are reviewed that may assist with advance the investigation of promising biomimetic materials in oral medicine.

## Search strategy

To identify appropriate studies concerning biomimetic materials for oral medicine, the literature search was carried out in the electronic databases without restriction to regions, or publication types. The primary sources were the electronic databases of Web of Science, Springer Link, and Science Direct. Until August 2023, these following Medical Subject Headings (MeSH) and non-MeSH terms were employed in the electronic search: “biomimetic enamel” or “biomimetic dentin” or “biomimetic tooth” and “biomimetic implants” or “biomimetic coating” or “tooth regeneration” and “guided tissue regeneration” or “GTR” or “biomimetic periodontal tissue” or “biomimetic cementum” and “biomimetic jaw” or “biomimetic bone” or “biomimetic jawbone”. If any of the next conditions were found, literatures were excluded: (1) non-English written, (2) published more than ten years ago, (3) the biomimetic mechanism, not the material. On the basis of above, this review's inclusion criteria included: (1) reviews, (2) animal, and (3) in vitro studies. The main information about selected literatures was then obtained and analyzed to present a summary of the biomimetic materials for treating tooth defect, tooth loss, periodontal diseases and maxillofacial bone defect.

## Biomimetic materials for treating tooth defect

Tooth defect is a disease that the shape and structure of tooth hard tissues are damaged. Tooth hard tissues are composed of enamel, dentin and cementum [16]. Typically, the acids from carbohydrates in the oral environment initially impact enamel, leading to its demineralization. As caries progresses, dentin demineralization occurs and aggravates, which even leads to further tooth defects [17]. If no measures are taken to stop this process, it will cause more serious oral diseases such as endodontics and periapical diseases. The common clinical treatment for tooth defect is direct restoration. Compared to conventional materials for tooth defect, the biomimetic remineralization materials can achieve the bottom-up reconstruction of enamel or dentin, which have better outcomes for caries prevention and treatment. Inspired by the protein-mediated mineralization process, biomimetic materials with similar functions as protein have been created to induce mineralization [18]. Nowadays, it is possible to realize the natural mineralization partly in vitro through imitating the mechanisms of biomineralization. Additionally, the biomimetic restoration materials provide a better choice for serious tooth defect or higher aesthetic requirements with the focus on the same hardness, stiffness and strength as human teeth.

### Biomimetic materials for treating enamel demineralization

Enamel is a highly mineralized tissue covering the surface of the tooth crown, which is the only cell-free tissue in the human body. As the hardest tissue in the human body, enamel contains large amounts of inorganic content. Most of its inorganic substances are Hydroxyapatite (HA)[Ca_10_(PO_4_)_6_(OH)_2_] containing calcium, phosphorus, carbonate, fluorine and other micronutrient [19]. Singular HA crystallite, ranging in thickness from 15 to 50 nm and width from 40 to 150 nm, grows along the C-axis with lengths of at least 100 µm. Multiple HA crystallites are arranged as prisms under the control of ameloblasts [20, 21]. In the transverse section, the special organized method of the prisms shows the characteristic “Fish scale structure” [22]. The change in HA crystal orientation and organic content decreases hardness and elastic modulus from the enamel’s surface to the bottom, while increasing toughness [23].

The bacteria, accumulating on teeth surface due to bad oral habits, orthodontic treatment and so on, can produce the acids, causing the decrease in pH [24]. It has been shown that the solubility of HA increases nearly 10 times for every 1 decrease in pH, resulting in enamel demineralization. Without the effective measures to intervene in time, the demineralized area will continue to expand, conspiring to tooth hypersensitivity and even develop into tooth decay [25]. Besides, enamel tissue contains no cells and cannot regenerate spontaneously once fully mineralized [26]. Fluorine-containing products are widely used for treating enamel demineralization among clinical practices. The fluorine ions bind to HA to form fluoridated hydroxyapatite (FHA), which have better acid resistance and remineralization than HA [27]. However, the risk of fluorosis, fractures and accidental ingestion in children cannot be ignored [28]. The materials with safer and more functional benefits need to be developed to prevent the dental caries. Inspired by the crucial substance in the enamel mineralization process, the biomimetic remineralization materials provide a new strategy for enamel reconstruction. Taking advantage of biomimetic approaches, considerable trails have been conducted to induce enamel remineralization. The biomimetic materials inspired by amorphous calcium phosphate (ACP) and enamel matrix proteins are summarized in this section with the main focus on the current development and limitations in the field.

#### Inspired by ACP

According to “Ostwald-Lussac Law of Stages”, as the basic metallogenic unit in the formation of biomineralization, ACP provides the necessary calcium and phosphate ions. It was shown that ACP can nucleate and form HA under certain conditions, thus forming new enamel [29, 30]. Casein phosphate polypeptide (CPP)-ACP, chitosan (CS) and triethylamine (TEA) not only stabilize ACP by balancing supersaturated calcium phosphate (CaP) ions in the oral environment, but also remineralize enamel in situ [31–33].

The motif sequence of CPP is similar to the salivary protein “statherin”. It can adsorb calcium and phosphate ions while combining with fluorine ions to form a stable soluble amorphous complex known as CPP-ACP [34, 35]. In addition to being used as pastes for the non-invasive treatment of white spot lesions, CPP-ACP may be added as an ingredient in toothpaste, yogurt or gum, achieving both preventive and commercial value [36–38]. Tin is confirmed to be comparable to fluoride, which promoted the binding of CPP complex to calcium ions [39]. When CPP-ACP is stabilized by SnF_2_, through the interaction and cross-linking mechanism, the nanofilament coating is assembled on the tooth surface. The coating enhances the mineral combination with better mineralization ability. However, the remineralization induced by CPP-ACP still exist several limitations. The rapid and uneven release rate of ACP precursor contributes to only partial mineralization but not a mineral layer [40, 41]. One way to solve this problem is to build a new delivery system for even release of mineralized precursors periodically. A delivery system (PAA-ACP@aMBG) established from aminated mesoporous bioactive glass (aMBG) could realize the even releases [42]. The ACP in the system was stabilized by the polyacrylic acid (PAA). The results showed that the remineralized layer could reach a thickness of 62.56 ± 4.98 μm and was similar to CPP-ACP in terms of hardness as well as color. Besides, the capability to release ions was related to the change of oral pH, which was suitable for oral environment.

Chitosan is a linear chain polysaccharide, which is obtained from chitin by deacetylation. It can store the ions required for mineralization, immobilize the HA, stabilize ACP in acidic environment as well as form a barrier with etching enamel surface to prevent further demineralization [33, 43–45]. A chitosan-agarose polysaccharide-based hydrogel has been fabricated, which can form biomimetic growth layer without any gaps [46]. The Ca/P value of the hydrogel is close to that of the native enamel and has the formation of enamel-like hierarchical structured layers under.

As scientific observation techniques advance apace, based on observations of zebrafish fin bone and nacre growth [47, 48], biomineralization has been found to be a process of epitaxial growth of crystals on an amorphous mineral layer [49]. Therefore, a rational design of HA and ACP structures has been presented that enable materials to simulate the induction of biomineralization front and realize the epitaxial development of enamel [50]. Notably, the smallest ACP particles, calcium phosphate ion clusters (CPICs), are not stable, and cannot spontaneously coalesce plus nucleate [31]. In order to stabilize CPICs, TEA was added, and its regulated removal could contribute to the creation of pure HA. The outcomes demonstrated that the mechanical characteristics of the mineral layer generated by the biomimetic material were almost identical to those of natural enamel. Additionally, the “Fish scale structure” that replicated natural enamel was observed, but the thickness was only 2.8 μm. This facilitated precise reconstruction from the nano-to the macro-scale.

#### Inspired by enamel matrix protein

Enamel matrix proteins (EMPs) play a pivotal role in the development of enamel [51]. EMPs are mainly composed of the combination of amelogenin (AMEL), enamelin, ameloblastin, of which AMEL is more than 90% [52]. AMEL has three domains, two of which are particularly essential: the hydrophobic N-terminal domain and the hydrophilic C-terminal domain. The former can be combined with apatite [53], and the latter has the ability to induce ACPs to transform into ordered crystals [54]. Studies have shown that AMEL can self-assemble into nanosphere near the HA [55]: the C-terminal exposed, N-terminal internally stable ACP [56], forcing HA growth along the C-axis and inhibiting crystal-crystal fusion [55]. Thus, biomimetic mineralization of enamel based on AMEL has been carried out. However, due to its propensity for denaturation and contamination, extraction and purification are challenging processes. Polypeptide biomaterials are commonly used in experiments instead of AMEL for their stability and ability to imitate the functions of natural proteins.

32 particular amelogenin-derived peptides (ADPs) in the (180 amino acid-long) amelogen (rM180) were detected by the new development of bioinformatics scoring matrix [57]. It turned out that the 22-amino acids long peptide ADP5 can best express the unique properties of AMEL. Under calcium and phosphate ions conditions, the research group has been able to build a crystalline mineral layer over enamel lesions with the help of a shortened 22-amino-acid long amelogenin-derived peptide (shADP5). The morphological characteristics similar to that of healthy enamel can be discovered in the newly formed crystalline mineral layer under the condition of low concentration fluoride. But its hardness and elastic modulus are slightly lower for the thickness is only 1–2 μm.

It is not difficult to see that the biomimetic enamel formed by AMEL analogue alone is not ideal in terms of mechanical properties. On the basis of literature data, the addition of enamelin was carried out in an effort to improve the nucleation rates [58]. As a result, other EMPs paired with AMEL to build a biomimetic environment are expected to have optimal mechanical properties. Meanwhile, leucine-rich amelogenin peptide (LRAP), which retains the charged N- and C- terminal of full-length AMEL, has also been confirmed to play the role of enamel mineralization in place of AMEL [59–61]. Not only can LRAP self-assemble into nano-spheres on the basis of enamel development [60, 62], but it can also promote the differentiation of ameloblasts or odontoblasts in vitro [63]. Therefore, an innovative design is to fabricate biomimetic EMPs using modified LRAP and non-amelogenin analog (NAA) to build a microenvironment [64]. The enamel-like tissues with prismatic and interprismatic structures were regenerated, whose mechanical properties are similar to those of tooth enamel. The regenerated mineral layer is around 2 μm thick.

In summary, even though the regenerated layers of various materials mentioned above can be similar to or even better than enamel concerning microstructure and mechanical properties, their thickness is still limited to micrometers. This limitation hinders the application of biomimetic materials that induce enamel remineralization to enamel lesions. If a breakthrough in thickness can be made, it is believed to have broader applicability for biomimetic materials in tooth defect.

### Biomimetic materials for direct restoration

Dentin is composed of collagen matrix, well-ordered nano-hydroxyapatite crystals and a small amount of non-collagen protein [18]. It has a higher organic matrix composition than enamel, accounting for 30% of the total weight of dentin [65, 66]. About 90% of the organic matrix is collagen (mainly type I collagen) and 10% is non-collagenous protein (NCP) [67, 68]. When caries progress to dentin, it will cause dentin demineralization as well as type I collagen fiber exposure. Under the circumstances, Patients may experience symptoms such as sensitivity and pain. The conventional treatment is to operate direct restoration after removal of caries. However, the using of acid etchant may cause the collapse of dentine reticulum, incomplete penetration of the resin, and interfacial microleakage [69]. When the pH of interface drops due to microleakage, the collagenase is activated along with the exposed type I collagen is degraded [70]. This process leads to the instability of the resin-tooth bonded interface, which eventually conspires to secondary caries. Considering these risks, making the bonding interface more stable is necessary. For this purpose, biomimetic remineralization materials have a promising development prospect. Through imitating the essential process and structure of dentin mineralization, the bottom-up induction of dentin remineralization can achieve the reconstruction of dentin, which can enhance the anti-enzymolysis capabilities, improve the durability of adhesives to bond, in order to prevent secondary caries effectively [71].

**Fig. 1:**
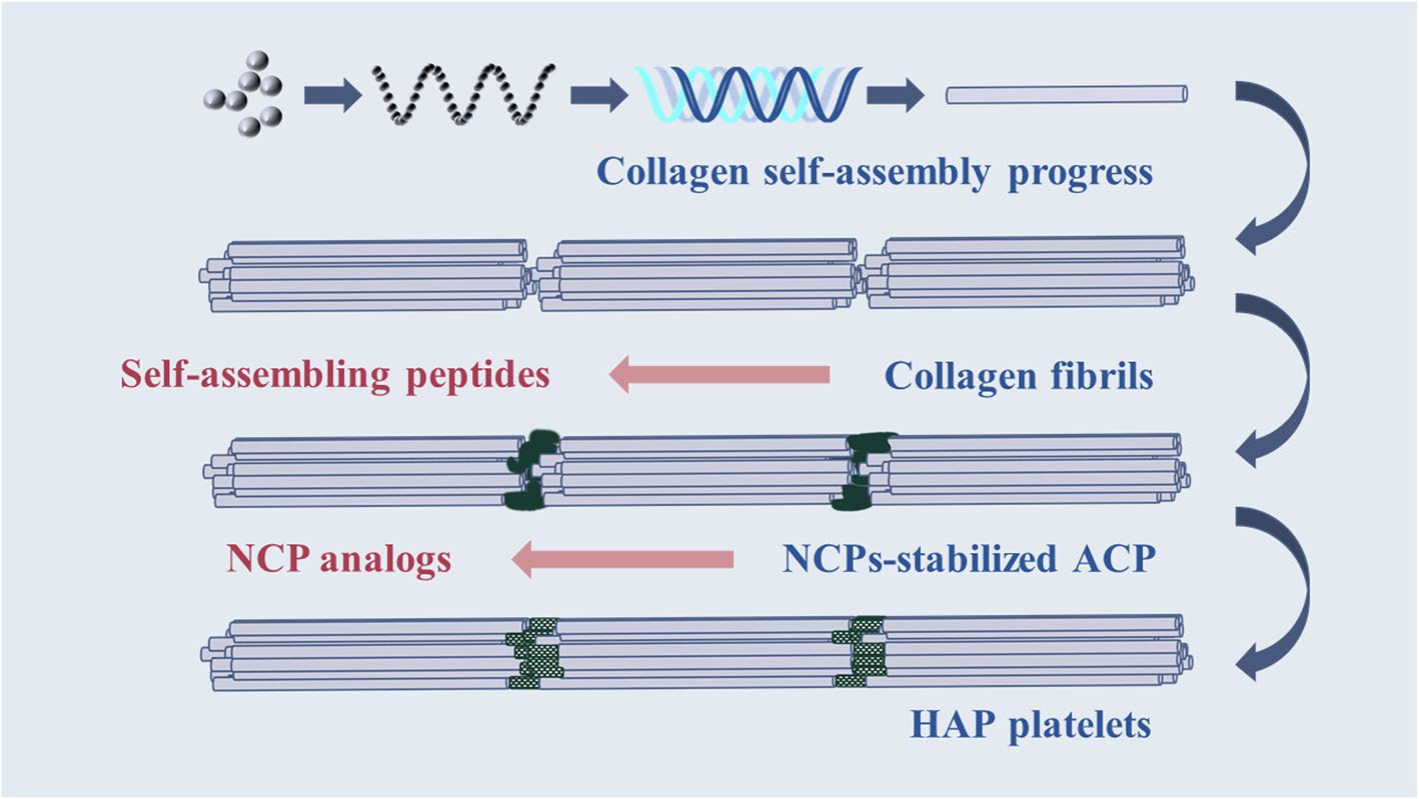
The intrafibrillar mineralization process of human dentin and the biomimetic materials inspired by the important components in this process

Dentin mineralization can be divided into intrafibrillar mineralization and extrafibrillar mineralization by location. Since it has been demonstrated that the intrafibrillar mineralization is crucial for the dentin's nanoscale mechanical properties, this mineralization has been a main focus [72]. The mineralization mechanism of dentin fibers is very complex, and the most widely accepted one is illustrated in Fig. 1. Firstly, type I collagen forms collagen fibers through the process of cell secretion, aggregation as well as self-assembly [73]. Then, ACP penetrates into collagen fibers followed by a conversion into hydroxyapatite crystals (HA crystals) under the regulation of NCP [74–76]. Next, HA crystals grow and complete the mineralization. In this process, collagen fibers provide the mineralized scaffolds along with/together with nucleation sites, while NCP mainly plays a role in stabilizing plus guiding ACP [77]. In general, the biomimetic remineralization materials are based on the inspiration of important substances in dentin mineralization mainly including collagen and NCP, as shown in Table 1.

**Table 1:** Dentin biomimetic mineralization materials

biomimetic inspiration	Biomimetic mineralization materials	category	Main findings	Reference
**Inspired by collagen**	PA	Active peptides	Self-assemble and induce HA to grow along the long axis of fiber structure	[78, 79]
	MDG1	Active peptides	Self-assemble and direct the production of HA	[80]
	MPP3	Active peptides	With two more stable peptides and two opposite charged residues, the mineralization rate is faster	[81, 82]
	P_11_-4	Active peptides	Self-assemble and increase the hydrolytic resistance of type I collagen fibers with no cytotoxic effects to odontoblast-like cells	[83–88]
	ID-8	Active peptides	Induce intrafibrillar mineralization	[89]
**Inspired by NCP**	8DSS	Active peptides	Induce extrafibrillar mineralization	[90–93]
	SSD3	Active peptides	Induce intrafibrillar mineralization	[94]
	PASP	polyanions	Form apatite crystals in the re-mineralized liquid	[75]
	PAA	polyanions	Prevent ACPs from accumulating and mineralizing outside collagen fibers	[95, 96]
	PVPA	polyanions	Phosphorylate collagen fibers	[97, 98]
**Inspired by collagen and NCP**	PAMAM	Dendrimers	Self-assemble and induce both intrafibrillar mineralization and extrafibrillar mineralization	[99–102]
	PAMAM and NACP	Dendrimers	Have long-term remineralization capability	[103]
	PAMAM and MMP inhibitors	Dendrimers	Inhibit MMP and stabilize collagen	[104]

*Abbreviations*: *PA* peptide-amphiphile, *MDG1* mineral directing gelator, *MPP3* PGEKADRAEKADRA, *P*_*11*_*-4* CH_3_CO-Gln-Gln-Arg-Phe-Glu-Trp-Glu-Phe-Glu-Gln-Gln-NH_2_, *ID-8* Ile-Asp-Ile-Asp-Ile-Asp-Ile-Asp, *8DSS* eight aspartate-serine-serine, *SSD3* three ser-ser-asp, *PASP* poly aspartic acid, *PAA* polyacrylic acid, *PVPA* polyvinyl phosphonic acid, *PAMAM* polyamidoamine, *NACP* nanoparticles of amorphous calcium phosphate, *MMP* matrix metalloproteinases

#### Inspired by collagen

Collagen is one of the indispensable organic components during the biomineralization process, providing three-dimensional scaffold structure and nucleation sites. Early studies have shown that the most straightforward way for biomimetic mineralization is to induce the formation of mineral crystals directly by using collagen [105, 106]. The difficulty in obtaining and preserving collagen limits the implementations of this approach. Therefore, self-assembling materials have emerged and gained attention. The most classic one is peptide-amphiphile(PA) synthesized by Stupp et al. [78]. Subsequently, Hartgerink et al. [79] found that the PA could self-assemble into nanofiber structure in solution. The phosphoric acid groups and carboxyl groups on the periphery of the fibers can combine with calcium ions, thus inducing HA crystals to grow along the long axis of fiber structure. This process partly simulates dentin mineralization. A set of self-assembling peptides was designed, which could foster the growth of HA crystals along the long axis of fibers [80, 81]. The self-assembling peptides have also been investigated as luting agents for indirect restorations, especially mineralization of the adhesive interface has been achieved [82]. Moreover, Self-assembling peptide (P_11_-4) was created that could self-assemble into collagen fibers by layering [83]. Currently, P_11_-4 has been extensively studied for its capabilities to prevent collagen proteolysis in dentin and induce mineralization of HA crystals [84–86]. Hence, P_11_-4 has remarkable potential in the field of dentin remineralization [87]. However, the majority of P_11_-4 are investigated in vitro, thus it is hoped that additional studies in vivo clinical will be conducted in the future to demonstrate its long-term stability in oral environment [88]. Notably, another self-assembly β-sheet peptide ID-8 (Ile-Asp-Ile-Asp-Ile-Asp-Ile-Asp) could serve as the template for intrafibrillar mineralization, which led to the preservation of calcium inside collagen and considerably improved collagen's hydrophilicity [89].

#### Inspired by NCP

NCP is known to play an indispensable role in dentin mineralization by stabilizing ACP and inducing intrafibrillar mineralization. There are many kinds of NCP, of which dentin phosphoprotein (DPP), as well as dentin sialoprotein (DSP), is the most significant [107–109]. DPP has the function of mineralization nucleation, while DSP is responsible for inhibiting the peritubular dentin deposition and preventing the dentin tubules closure [109, 110]. The majority of current research focuses on DPP, whereas DSP lacks relevant research. Inspired by the multiple repeatable amino acid sequences (aspartate-serine-serine, DSS) in human DPP, eight tandem DSS sequences (8DSS) have been found that have the ability to promote dentin remineralization [90, 91]. This ability has been demonstrated in fully demineralized dentin [92]. Besides, 8DSS is able to maintain efficient dentinal tubule occlusion even after acid challenge, which shows potential in treating dentin hypersensitivity [93]. Also inspired by DPP, another repeatable amino acid sequences, three tandem SSD (Ser-Ser-Asp) sequences (SSD3) were designed. It was found that 80% of Ser could be phosphorylated by casein kinase [94]. The test showed that phosphorylated SSD3 could induce intrafibrillar mineralization. DPP is rich in aspartic acid, so polyaspartic acid (PASP) has attracted much attention. PASP has a large amount of negative charge, which is equipped to stabilize ACP [75, 111]. While having plenty of carboxyl groups, PAA can stop ACPs from accumulating and mineralizing outside of collagen fibers [95]. Based on these, the creation of the polymer-induced liquid-precursor (PILP) technique is worth mentioning [96]. PILP employs anionic polymers, such as PAA and PASP, to stabilize ACPs and promote intrafibrillar mineralization. Its efficacy was assessed, which indicated that this technique could feasibly induce dentin remineralization [112]. Currently, due to the important functions of strontium in bone, scholars have devised a strategy for gathering collagen with Sr-doped HA through the PILP process, which may provide a new choice to realize dentin remineralization [113]. In addition, polyvinylphosphonic acid (PVPA) contains phosphate groups that can phosphorylate collagen fibers, so PAA is often used in combination with PVPA [97, 98].

Polyamidoamine (PAMAM) molecule has been widely studied in the field of biomimetic mineralization due to its excellent chemical and biological properties [99]. To some extent, PAMAM can be used as both an NCP and a template for remineralization [100, 101]. The design of carboxy-containing PAMAM molecules has achieved dentin remineralization both in vitro and in vivo [101, 102]. PAMAM and nanoparticles of ACP (NACP) have been combined in some recent researches to create novel materials with the ability to remineralize, indicating that this combination may have long-term remineralization potential [103]. It's worth noting that the degradation of collagen fibers may result from matrix metalloproteinases (MMP) activation. So, PAMAM materials containing MMP inhibitors have been created, which have the ability to stabilize collagen [104]

### Biomimetic materials for indirect restoration

**Fig. 2:**
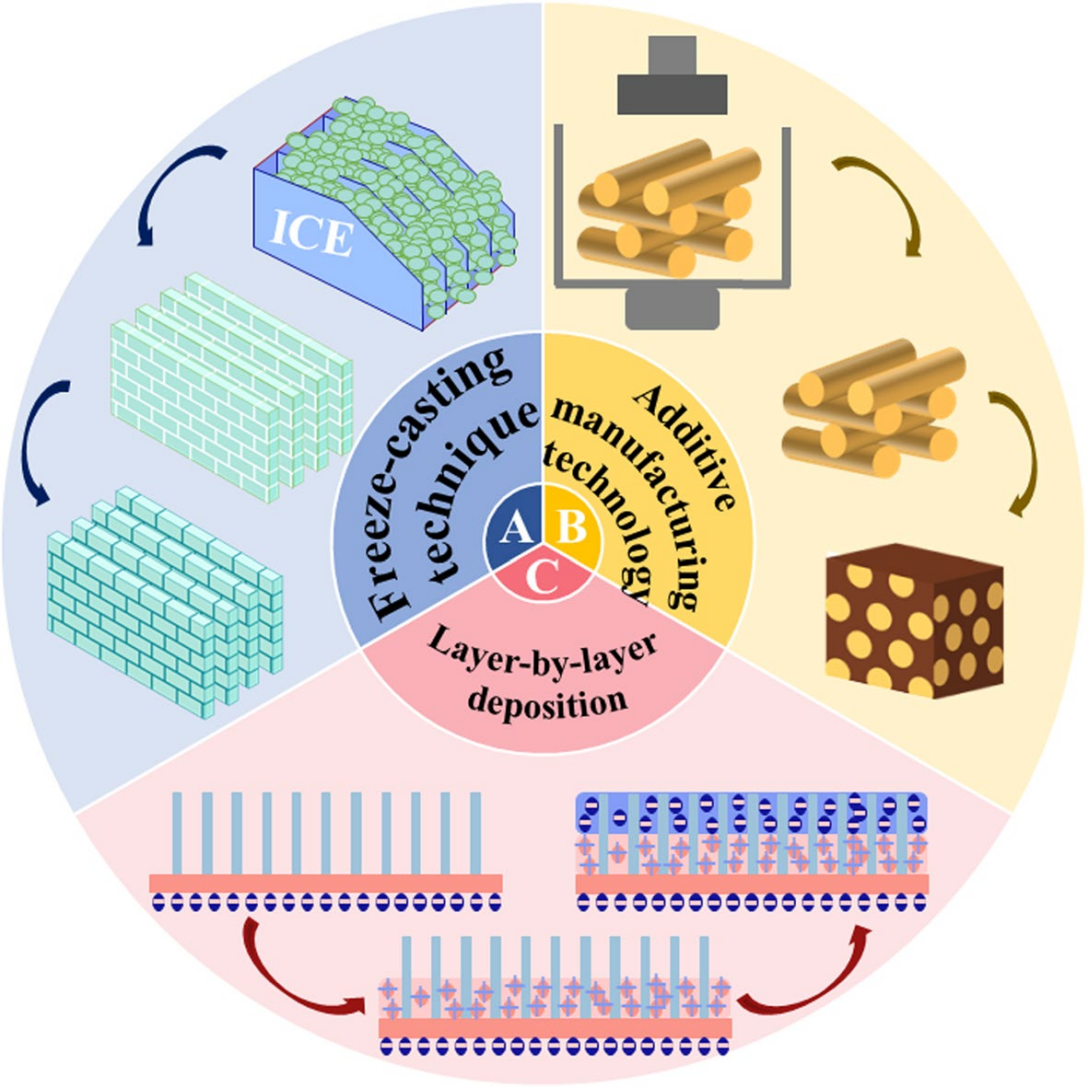
Schematic diagram of biomimetic indirect restoration materials preparation

When the tooth defect is serious or needs to achieve a higher aesthetic requirement, artificial restorations are required for indirect restoration. Among various artificial restoration materials, 3 mol% yttria-stabilized tetragonal zirconia polycrystalline (Y-TZP) materials occupy a prominent position on account of their high hardness, strong stiffness, excellent biocompatibility, and satisfactory aesthetic effect [114, 115]. However, Y-TZP is nearly four times harder than human tooth enamel, which may damage the antagonistic teeth [116]. In addition, due to its brittleness, it may cause adverse factors such as cracks [117]. Thus, it is necessary to use biomimetic materials to avoid the corresponding damage. As an alternative to tooth tissue (enamel and dentin), artificial restorations ought to have properties resembling those of tooth tissue in order to replicate their biomechanical functions. The ideal restoration material must have a similar hardness, stiffness and strength as human teeth. Meantime, good anti-fatigue performance cannot be overlooked, which makes artificial restorations possible to use in the oral environment safely and efficiently for a long time. As shown in Fig. 2, freeze-casting technique, additive manufacturing technology and layer-by-layer deposition have all been applied in the fabrication of biomimetic restoration materials. We will elaborate on the different properties of biomimetic materials manufactured by various technologies.

#### Freeze-casting technique

Freeze casting is a new manufacturing technology for porous materials, which can be applied to composite materials simulating nacre structure [118, 119]. This kind of composite material has excellent mechanical properties, especially in toughness [120].

Inspired by the nacre structure of mollusk shells, a novel ceramic-polymer composite was demonstrated [121]. The composites were realized by using polycrystalline tetragonal zirconia stabilized with 3Y-TZP as scaffolds, then mixing with polymethyl methacrylate (PMMA) to produce nacre-like and brick-and-mortar composites with different mineral contents. The Young’s modulus and hardness of the composites are similar to that of enamel and dentin respectively due to different constitution and structure. Furthermore, materials can dissipate mechanical energy during cyclic loading, and significantly reduce the abrasion of antagonistic teeth [122].

Moreover, freeze casting may achieve accurate replication of the complex layered structure of enamel. Based on amorphous intergranular phase (AIP, Mg-substituted ACP), a new synthesis route of multi-scale enamel analogues was designed [123]. The enamel-like aligned lamellar composites were created utilizing a bidirectional freeze casting process on a polydimethylsiloxane (PDMS) wedge with AIP-coated HA nanowires/polyvinyl alcohol (PVA) slurry. The material is superior to other materials in stiffness, hardness, strength, viscoelasticity and toughness. As the most similar enamel-like material, it is expected to be a new generation of dental restoration material.

#### Additive manufacturing technology

The freeze casting technology is still limited in the production of complex geometric ceramic composites [121, 124], while additive manufacturing (AM) has great potential in this aspect [125]. AM has been widely applied in multiple fields for its ability to manufacture a variety of materials, such as metals, ceramics, polymers, materials of biological origin, etc., is widely developed in various fields [126, 127]. Because of its flexibility in designing materials, it is especially suitable for studying the structure and mechanical properties of materials [128].

The nacre-inspired ceramic composites described above have demonstrated excellent properties, but their load-bearing capacity is compromised by stress concentration at the hard/soft interface caused by discontinuous ceramic phases [129]. Scholars have discovered a new bi-continuous phase ceramic composite by studying the impact surface of the mantis shrimp's hammer-like dactyl club to improve the load-bearing capacity [130]. A continuous ceramic scaffold triply periodic minimal surface (TPMS) structure was fabricated by a digital light processing (DLP) printer, which mimics the bi-continuous structure of mantis shrimp [131]. Subsequently, the epoxy polymer is injected into the scaffold by polymer infiltration, resulting in a material with a high Young's modulus and hardness. The long-term clinical application will be safer and more comfortable for its low density and satisfactory biocompatibility. It was shown that the material with excellent energy absorption characteristics has better load-bearing capacity and extraordinary toughness [131].

Robocasting can create connected grids of ceramic rods that are placed perpendicularly to the occlusal surface to replicate the properties of natural materials. Inspired by the microscopic structure of enamel, a biocompatible polymer is injected into printing an open HA rod network to produce ceramic/polymer dental composites [132]. Although the biaxial strength, as well as the wear resistance of the composites, are close to requirements, some features are still insufficient. However, it is believed that with optimization, this material has great potential for usage in new types of dental composites with novel mechanical energy reinforcement and higher durability.

#### Layer-by-layer deposition

The techniques mentioned above rarely mimic the basic prism-like structure of enamel, on the contrary, layer-by-layer (LBL) technology performs well in this connection. The LBL technique, first proposed by ILER in 1966 [133], works by alternately attracting polyelectrolyte matrix with opposite charges onto a substrate to produce a multilayered structure [134]. LBL technology can control the hierarchical structure, multilayer interface and morphology of multilayer materials at the nano level [135–137], which has a great influence on the physicochemical properties of materials [134, 138].

In the study of Yeom et al. [139], the ZnO nanowires were hydrothermally fabricated first and then alternately attracted polyallylamine (PAAm) and PAA by LBL deposition method to successfully produce (ZnO/LBL) composites. The composite materials' viscoelastic figures of merit (VFOM) and weight-adjusted VFOM were over conventional material limitations of 0.6 and 0.8, which are similar to those of enamels. While providing lower materials density (ρ), the material is cost-effective to produce, opening a new avenue for the design of lightweight materials with load-bearing, vibration and aging resistance. By imitating the enamel-dentin junction, β-FeOOH nanocolumns were synthesized by hydrothermal method to begin with. Later on, the team used LBL technology to alternately immerse nanocolumns into tannic acid (TA) and PVA solution to create a tooth replicate with an interdigitated interface [140]. After mechanical testing, the elastic modulus (94 ± 10 GPa) and hardness (6.4 ± 0.8 GPa) of the material behave well. Additionally, it performs admirably in the areas of plastic dissipation energy, bending resistance, self-healing ability, bactericidal capability along with other aspects. And most importantly, the material possesses basic characteristics of enamel and dentin: viscous-elastic–plastic.

## Biomimetic materials for treating tooth loss

Tooth loss is a common oral disease, which can be caused by tooth agenesis, dental caries, periodontal disease, trauma and so on [141]. The loss of teeth not only affects people's speech, facial contour, mastication and other physiological functions [142, 143], but can generate epilepsy, heart disease, peripheral vascular disease as well [144–147], seriously damaging the physical and mental health of patients. However, there are only two dentitions during a human lifetime. The first is the deciduous dentition. As we age, permanent teeth replace deciduous teeth, forming permanent dentition [148]. When permanent teeth are lost, the human body cannot grow its own teeth to replace them. Therefore, the treatment of tooth loss has always been the focus of the research, and the relevant measures can be traced back to BC [149]. In the modern medical system, fixed partial dentures, removable partial dentures as well as dental implants are mostly applied in clinic [150–152]. However, the employment of dentures can conspire to bone resorption in patients, which can bring discomfort even unable to play physiological functions [149]. Furthermore, implantation failures are not unusual due to various factors, bringing trauma to patients [153]. Consequently, improving the success rate of implantation by studying biomimetic implants or using biotechnology to achieve functional tooth regeneration is very promising alternatives.

### Surface modified biomimetic dental implants

**Fig. 3:**
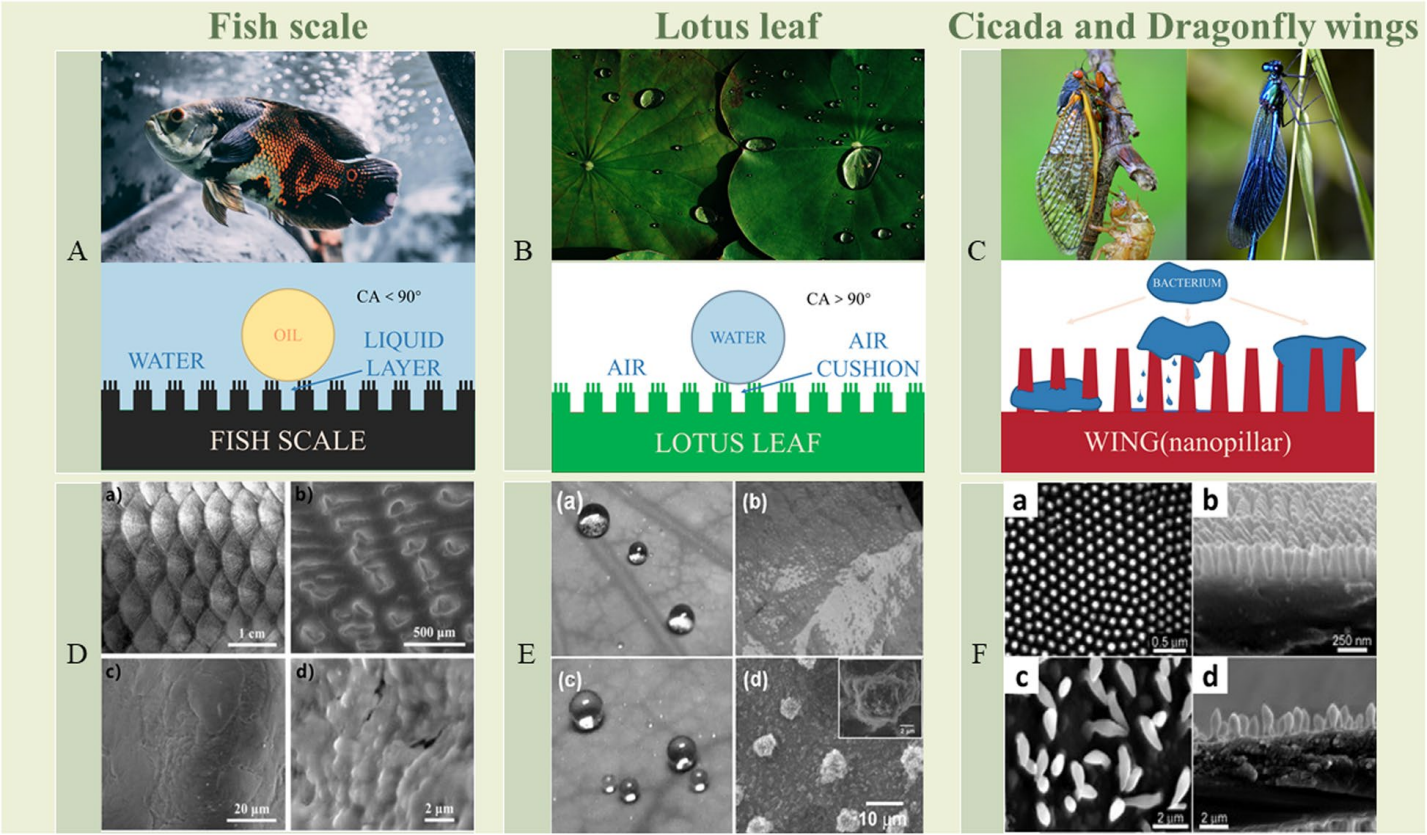
The inspirations of biomimetic multifunctional coatings and biomimetic nano-topography [159–161]

Infection has always been a major factor in implant failure, while the primary treatment in the past was using drugs and antibiotics [154, 155]. However, the increasing use of medications and antibiotics has resulted in the emergence of drug-resistant bacteria, which poses a serious threat to implant's longevity and even the overall health of body [156].To address this problem, the application of biomimetic modified surface on implant has received extensive attention. Biomimetic modified surface mainly includes biomimetic multifunctional coating and biomimetic nano-topography currently. Besides, the former mainly mimics the super-hydrophilic structure on the surface of fish scales and the superhydrophobic structure on the surface of lotus leaves, while the latter mainly focuses on the nanostructures on the surface of cicadas and dragonfly wings [157]. The inspirations and specific biomimetic materials can be seen in Fig. 3 and Table 2. The modified biomimetic surface on implant has antibacterial and bacteriostatic effects, which can improve the efficiency of bone integration, so as to increase the survival rate of the implant and prolong its life. In particular, the use of biomimetic surfaces could reduce dependence on antibiotics and slow the evolution of resistant bacteria at the same time [158].

**Table 2:** Surface modified biomimetic materials applied in implants

biomimetic inspiration	Property	Material(s)	Category	Major Upside(s)	Major downside(s)	Reference
fish scales surface	super-hydrophilicity	PEG	polymers	Anti-fouling, bacteriostasis, after adding cell adhesive sequences, it has favorable biocompatibility and bone binding	A risk of polymer degradation with time	[162]
		AMP	peptides	Anti-fouling, bactericidal capability, low cellular cytotoxicity, low antibiotic resistance	Structural complexity, expensive	[163]
lotus leaf surface	super-hydrophobicity	Silane	Silicon based materials	Bactericidal capability, osseointegration performance	—	[164]
Wing-shaped nanopillar structure of Cicada or dragonfly	bactericidal capability, self-cleaning property	Ti	metal	Bactericidal capability (S. epidermidis), hydrophobicity, low antibiotic resistance	—	[165]
		TiO_2_	metal	Higher bactericidal capability (E. coli and S. aureus)	—	[166]
		ZnO	metal	Bactericidal capability (E. coli and S. aureus)	—	[167]
		FHA	Ceramic	Bactericidal capability, hydrophilic, good osseointegration	—	[168]
		bSi	Silicon substrate	Highly hydrophobic, bactericidal capability (P. aeruginosa and S. aureus)	Sterilization was less effective than previously reported	[169]
		SiO_2_	Silicon substrate	Higher bactericidal capability efficiency	—	[170]
		PET	polymer	Bactericidal capability (E. coli and Klebsiella pneumoniae)	—	[171]
		PS-block-PMMA	copolymer	Dual bactericidal and bacteria-releasing function	—	[172]
		ZnO-PSBMA	Composite material	Dual bactericidal and bacteria-releasing function	—	[173]

*Abbreviations*: *PEG* polyethylene glycol, *AMP* antimicrobial peptide, *FHA* fluoridated hydroxyapatite, *bSi* black silicon, *PET* polyethylene terephthalate, *PS-block-PMMA* polystyrene-block-polymethy lmethacrylate, *ZnO-PSBMA* ZnO-poly sulfobetaine methacrylate

#### Biomimetic multifunctional coating

Infection is commonly following implantation. While biomimetic multifunctional coatings, which have recently become a trendy material, provide a choice to solve this problem. It mainly imitates the anti-fouling and bacteriostatic structure on the surface of fish scales and lotus leaves to inhibit the attachment of bacteria or directly destroy contaminating microorganisms [174]. The surface of fish scales has a super hydrophilic structure, and the hydration layer can effectively prevent the attachment as well as penetration of oil or bacteria [175]. Additionally, owing to the surface's super hydrophilicity, when the lotus leaf enters the liquid, a liquid–air layer will form between the hydrophobic surface and the liquid, inhibiting bacterial attachment and settlement [176]. The super-hydrophilicity and super-hydrophobicity are expressed by the contact angle (CA) of water droplets on the substrate. The CA of the former is < 90°, whereas the CA of the latter is > 90° [177]. In fact, bacteria are more inclined to adhere to slightly hydrophilic or slightly hydrophobic surfaces, and less adhesion occurs on super hydrophilic or super hydrophobic surfaces [178]. These two antifouling and bacteriostatic structures can be applied to implant coating materials to achieve similar functions [179].

Currently, implant coatings materials with super-hydrophilicity similar to fish scales mainly include polyethylene glycol (PEG), PEG polymers and antimicrobial peptides (AMP) [180]. PEG is probably the most widely used polymer for anti-bacterial adhesion on the surface of materials [163, 181]. However, its strong anti-adhesion also has an effect on eukaryotic cells, which inhibits the adhesion between implants and surrounding tissues. Therefore, the use of PEG usually requires the addition of cell adhesive sequences to maintain the normal adhesion of cells [162]. It can be combined and extended with various cell adhesive sequences, such as RGD (arginine-glycine-aspartate), silk sericin, etc. The latest research combined PEG with other polymers or fungicides with antiadhesive ability to form dual-function or even multifunctional antibacterial coatings, which could achieve better antibacterial effect. For example, scholars deposited TA and PEG on the surface of titanium and through a simple one-step method to form an anti-fouling coating at the same time, which can suppress the bacterial and platelet adhesion [182]. Moreover, TA was used as a bonding medium to combine inorganic hydroxyapatite nanoparticles (nHA) with organic PEG to form an organic–inorganic coating that has both antibacterial and osteogenic abilities [183]. In addition, PEG and CS could be combined to make antibacterial and bactericidal coating [184]. But this coating has not yet been applied in the field of oral medicine. Besides, another group investigated the effectiveness of the chitosan-based hydrogel coatings on the Ti_6_Al_4_V implants. It was discovered that this coating had strong dual-function antibacterial mechanisms including bacterial repulsion and contact killing, which also showed good biocompatibility in animal models [185]. Thus, this coating has broad application prospects in the field of implant materials. However, PEG still has many shortcomings. It has very poor stability and runs the risk of degradation with changes of environmental temperature and the passage of time, which will affect the coatings’ long-term effects [186, 187]. Facing the limitations of PEG, a new strategy was proposed: AMP. AMP was found in the salivary glands and was effective at killing bacteria such as Streptococcus mutans [188]. Besides, the combination of AMP and cell adhesive sequences can achieve bactericidal effect and maintain normal cells adhesion [162]. More than that, covalent immobilization of AMP has been extensively explored [189]. This approach is particularly effective for anti-infection of implants in combination with the following nanostructured surfaces [190]. It is worth noting that although AMP has a good bactericidal effect, it is still possible to proliferate on the implant surface in the presence of even few bacteria [191]. In order to enhance the bactericidal property, PEG, AMP and cell adhesive sequences were combined in a certain way to achieve bacteriostasis, sterilization and maintain normal cell adhesion [192–194].

Implant coating materials that mimic the super-hydrophobicity of lotus leaf mainly include silane. Usually, silane materials are used as “anchors”, which refers to the silanization of implant surface, especially titanium surface. During the silicification process, various molecules (such as peptides, polymers, proteins, etc.) are covalently attached to the surface of the implant [195]. While these attachments play their corresponding roles, previous studies have shown that the use of silane primer can reduce the surface energy of implants and improve hydrophobicity [196]. In addition, some silanes have also been found to have their own biological activity. For example, some scholars introduced the 3-aminopropyl triethoxysilane on the surface of titanium implant, and found that silane adhesion layer can promote angiogenesis and osteogenesis [197]. Besides, the triethoxysilylpropyl succinic anhydride (TESPSA) used on titanium implants had no negative effect on human fibroblast viability and had osteoinductive and antibacterial activity [164].

#### Biomimetic nanostructures

Faced with the major challenge of bacterial infection in implants, the studies of biomimetic nanostructures have attracted much attention. The biomimetic nanostructures mainly simulate the natural nanostructures of cicada and dragonfly wings to achieve similar bactericidal properties, while preserving the vitality of human cells at the same time. The surfaces of cicada and dragonfly wings are superhydrophobic and have important nanopillars (NPs) that can kill bacteria [198]. Previous research proposed three bactericidal mechanisms for NPs. First, the nanopillars directly pierce the cell membrane of the bacteria as adhesion forces to pull the bacteria towards the surface [168]. Second, when bacteria adhere to the edges of the NPs, they stretch the cell membrane and eventually break between the NPs [199–201]. Third, the adhered bacteria move through the column, leading the inner cytoplasmic membrane to separate [202]. These NPs also play a vital role in surface wettability (super-hydrophobicity), but due to their strong adhesion, bacteria are tightly attached to the NPs, so this wettability does not contribute to their bactericidal effect [203].

Besides, the dimensions of the natural NPs of cicada and dragonfly wings are within a certain range. Actually, the height, diameter and spacing of these NPs vary from species to species [204]. The main range of NPs on cicada wings is about 82–148 nm in diameter, 400 nm in height, 190 nm in spacing, and these NPs are closely arranged in hexagons [198, 205]. The nanostructures on the dragonfly wing are similar to that on the cicada wing, but is relatively irregular in shape, ranging from 83 to 195 nm in diameter. Compared with cicada wings, the tip diameter of the NPs on dragonfly wings is smaller, while the membrane thickness of gram-negative bacteria is thicker than that of Gram-positive bacteria. That is why the NPs on dragonfly wings are more likely to kill Gram-negative bacteria [157]. Hence, the small difference in the tip diameter of the biomimetic NPs has a significant impact on the bactericidal effect.

Based on the above findings, researchers have explored the size of NPs with the strong bactericidal effect. It was shown that among all the nanostructures, those with the NPs arrays with a height of 100–500 nm, a diameter of 10–300 nm, and a spacing of 10–380 nm had the stronger ability to kill bacteria [206].

Currently, the nanostructures of pure titanium surfaces in titanium materials show excellent antibacterial activity without gradually weakening with the release of antibacterial substances. Besides, it has no intrinsic cytotoxicity, and inhibits biofilm formation in vitro [165]. Then, a new surface technique containing polydopamine (PDA) and silver nanoparticle-loaded TiO_2_ nanorods (NRDs) coatings on Ti alloy was devised. It was shown that this method could not only pierce the bacteria physically but also release silver ions to enhance the antibacterial effect [166]. Among other metal materials, ZnO nanorods expressed high bactericidal activity against Staphylococcus aureus and Escherichia coli both in vivo and in vitro [167]. Furthermore, using Cap to make the FHA nanopillar is a way that saves time, effort and costs less. The FHA nanopillars have both bactericidal effect and osteogenic function, which are critical for dental and orthopedic implants [207]. Moreover, black silicon was the first NPs analogues [208]. And it was found that the NPs made of black silicon had favorable bactericidal activity against P. aeruginosa and S. aureus [169]. Interestingly, NPs made of silica were more efficient at killing bacteria than those made of black silicon [170]. In addition to metal and inorganic nonmetallic materials, there are also ones composed of polymer. For instance, NPs made by polyethylene terephthalate (PET) has the function of killing E.coli and K.pneumoniae [171]. Recently, researchers made NPs by placing a polystyrene-block-polymethy lmethacrylate (PS-block-PMMA) diblock copolymer on silicon substrate [172]. In particular, this structure has the dual function of killing bacteria and releasing dead bacteria. This dual function effectively solves the problem that the bactericidal effect of the NPs is ineffective due to the accumulation of dead bacteria. Analogously, there are some composite materials with dual function were designed. The ZnO and zwitterionic polymers have been combined to make composite materials, which not only can retain the bactericidal effect of the NPs, but also achieve the release of bacteria in the wet state [173].

### Tooth regeneration

With the development of stem cells and tissue engineering, tooth regeneration has become a hot topic in recent decades [209]. The theoretical basis of this study is tooth development, including its molecular regulation and cellular sources [210]. The approaches fall into two major classes as follows.

#### Whole-tooth engineering

This is the most ideal way to achieve tooth regeneration. By constructing artificial tooth embryo and simulating the physiological state of tooth growth, the biomimetic growth environment is built to achieve the purpose of functional tooth regeneration [210, 211].

Tooth development is a complicated process of interaction between epithelium and mesenchyme derived from cranial neural crest cells, and the factors expressed by epithelial cells are the keys to initiating tooth development [212]. In 2004, Ohazama et al. [211] conducted a landmark study in the field of whole tooth regeneration: they combined the mesenchyme gathered by non-dental stem cells with embryonic oral epithelium, and eventually found that the mesenchyme could be stimulated to produce an odontogenic response. When the combination was transplanted into the renal capsules of mature mice, the development of tooth structure and associated bones could be observed. In addition, the study discovered that the embryonic tooth primordia were transferred into the adult jaw, and ectopic teeth similar to the first molars concerning tissue were observed at the transplant site. This study confirmed that adult non-dental cells can create tooth primordia in vitro and grow into complete teeth in the human oral cavity for replacement by human transplantation.

In recent years, the current tissue engineering research on whole-tooth regeneration is still limited to animal experiments [213–215]. Besides, there are some problems that cannot be handled, such as the position, shape, and whether the regenerated teeth have the ability to replace the missing teeth to function. Therefore, tooth regeneration into human body application still has a long way to go. In order to achieve tooth regeneration as early as possible, given the close links between developmental biology and regenerative medicine [216], both constructing artificial tooth embryo and simulating biomimetic growth environment are inseparable from the research and exploration of tooth development mechanisms.

#### Root regeneration

Accounting for the previous discussion, whole-tooth regeneration research faces many technical problems. Tooth root is the basis for tooth function [217], and artificial crowns have access to be added on this basis. In order to achieve faster application of clinical, root regeneration has been carried on.

By building a biological scaffold in the shape of a tooth as well as selecting appropriate seed cells, a bio-root with a morphology, structure, and function similar to that of a normal tooth can be formed [218]. Different from the implant, the regenerated root has periodontal ligaments (PDLs). The PDLs are vital tissue structures involved in mastication, which are capable of transmitting the occlusal force to the brain and the whole body through the periodontal ligament mechanoreceptors. Due to the presence of PDLs, the masticatory movement and physiological load can be adjusted to avoid overload damaging the oral tissues and improve masticatory efficiency [219].

The basic components of bio-root construction include seed cells, signaling molecules and scaffolds [220]. Among them, scaffold materials are essential for the formation of bio-root [221]. In the initial research of bio-root, scholars used many scaffold materials, such as biphasic HA/tricalcium phosphate (TCP), polyglycolate/poly-L-lactate (PGA/PLLA), poly-L-lactate-co-glycolate (PLGA), glue-chrondrotin-hyaluronan-tri-copolymer (GCHT), and collagen sponge [218, 222–224]. But these materials are unsatisfactory in some respects, for example, insufficient strength, difficulty in supporting occlusal force, lack of controllability, and no odontogenic properties. So far, human treated dentin matrix (hTDM) has been mainly adopted as the scaffold material [225–227]. As well as establishing natural root morphology [226], this material has good biocompatibility. It is able to release crucial proteins related to tooth development and help to induce regenerated root formation [228–230]. According to the research test, when the length of the TDM bracket is 9.4 mm and the upper and bottom diameters are 4.9 mm and 3.4 mm, both the proper stress distribution and mastication performance can be achieved [231]. Despite that, the limited source of hTDM is not conducive to future clinical applications. Porcine TDM (pTDM) is expected to be used as an alternative material due to its wide range of sources and analogous shape to hTDM [232]. But as a heterogeneous origin, pTDM inevitably brings about local inflammation in the body reaction [229]. Consequently, it is necessary to keep exploring pertinent immunological regulation.

## Biomimetic materials for treating periodontal diseases

**Fig. 4:**
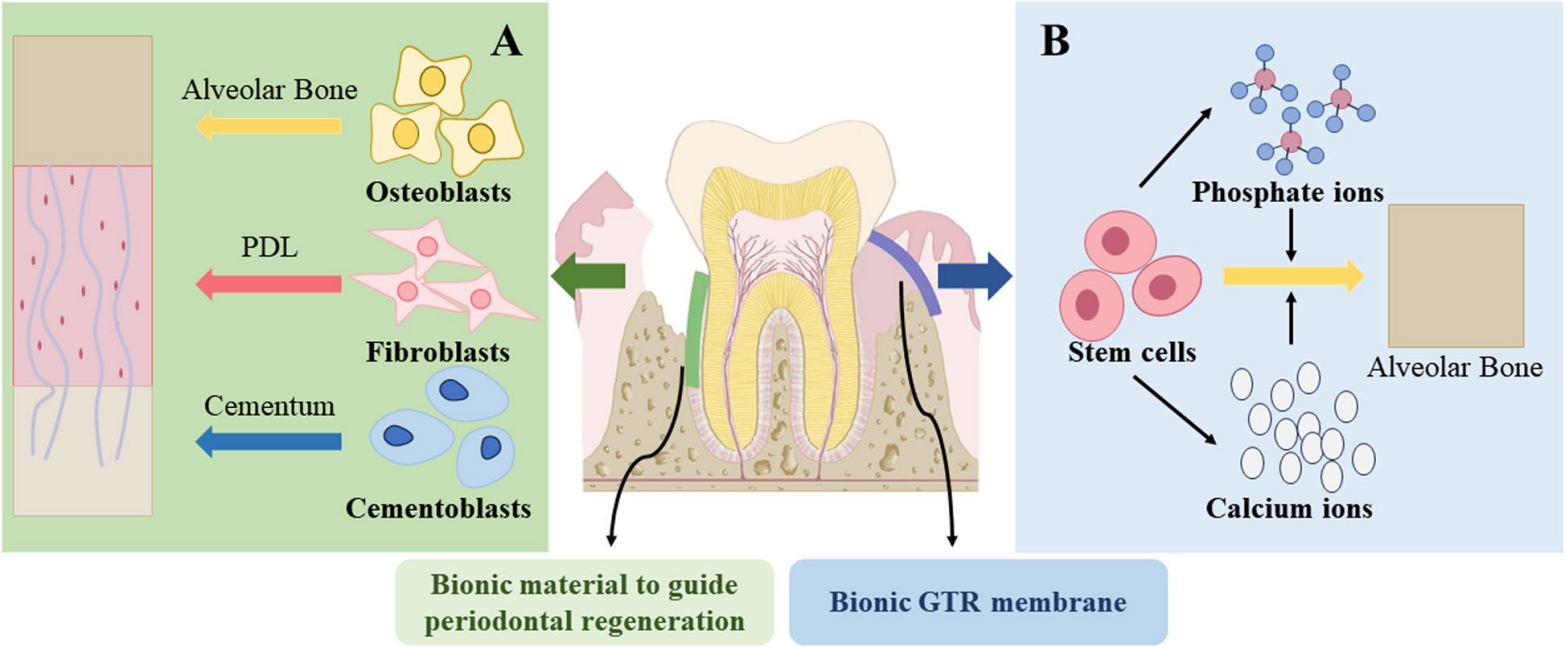
Biomimetic materials for periodontal tissue regeneration

Periodontal disease is a widespread oral condition, mainly manifested as gingivitis and periodontitis [233]. Alveolar bone and PDL are not damaged in patients with gingivitis, while periodontitis can cause the loss of the periodontal supporting tissue, periodontal pocket formation, attachment loss and bone resorption, ultimately leading to tooth loosening and loss [234, 235]. Therefore, the reconstruction of the lost supporting tissue is the ultimate goal in the treatment of periodontitis [236]. Since the advent of GTR technology [237], it has been brought into focus. The basic method is using a barrier membrane to block the growth of both gingival epithelium and connective tissue in the root surface during periodontal surgery. Simultaneously, inducing the PDL cells with regenerative ability to preferentially reach the root surface, thus forming new periodontal tissue (alveolar bone, PDL, and cementum) [237, 238]. However, traditional GTR, which relies more on the self-growth of PDL cells, hasn't always produced the best outcomes when treating periodontitis [239]. Hence, the application of biomimetic materials to improve the success of periodontal tissue regeneration has been brought up. The effect of biomimetic materials on periodontal tissue regeneration is shown in Fig. 4.

According to the above discussion, biomimetic barrier membrane is regarded as a delivery carrier or surface modification to promote the high-quality regeneration of periodontal soft and hard tissue in the damaged area [240].

Mussels in nature have good adhesion capacity in wet conditions. PDA is devised as a coating, which mimics the property inspired by mussels. Using electrospinning technology, PDA was applied to nanofiber membranes [241]. The coating demonstrated outstanding adhesive properties in this instance, promoting cell adhesion and accelerating HA mineral deposition in a simulated environment. At the same time, the team used a morphological micropattern approach to create micro patterns on the surface of the nanofiber membrane to locate cell adhesion and manipulate the structure of the membrane. The application of PDA coating not only promotes the regeneration of periodontal tissue but also prevents infection by prolonging the action time of effective substances. It is hoped that the use of this biomimetic material will spread to other fields that require drug delivery and healing techniques.

In GTR technology, cementum regeneration is considered to be a key [242]. Cementum is the hard tissue of the tooth that covers the root surface [243]. The major components of cementum are fluorine-containing nano-hydroxyapatite (nFHA) and type I collagen [244, 245]. Generally, acellular cementum and cellular cementum are classified according to the presence or absence of cells in the tissue [246]. Acellular cementum is a kind of primary cementum, whose main function is to provide attachment of teeth to periodontal tissues, and fix teeth [247]. Cellular cementum is a kind of secondary cementum, mostly located on the surface of acellular cementum. Cellular cementum plays an adaptive role in tooth occlusion to maintain tooth position, and its cementum cells can generate secondary cementum to compensate for abrasion, attrition and tooth displacement [248, 249]. In addition, it can coordinate with other periodontal tissues to regulate and disperse masticatory force [250]. Therefore, cementum regeneration is of importance among GTR.

The ideal regenerated cementum is very similar to the acellular extrinsic fiber cementum (AEFC), because the collagen fibers contained in AEFC belong to the extrinsic fibers connecting PDL, which are competent to maximize attachment function [251].

Biomimetic materials of cementum are primarily explored from two perspectives: replicating the biological development or stimulating the physiological structure.

Inspired by the process of cementum formation, a new type of biomimetic cementum by combining bioskiving and fluorine-containing biomineralization was constructed [245]. The alternative collagen lamellae made by bioskiving is a highly biomimetic twisted plywood model of cementum, which can be mineralized by adding it into the fluorine containing ACP solution stabilized by carboxymethyl chitosan (CMCS) to produce a biomimetic cementum material. The material can not only simulate the composition and structure of cementum but also promote the biological activity of periodontal ligament cells and induce the regeneration of cementum.

The “cell sheet” technique has great potential for the study of regenerative medicine cells [252]. While there are many approaches to make a cell sheet, the common mechanism is to apply different stimuli to cells in culture to establish cell–cell interactions that form cell sheets and their extracellular matrix (ECM) [253]. As technology advances leaps and bounds, cell sheet engineering has been proven to be useful for periodontal tissue regeneration [254], but the formation of collagen fibers in cementum remains a challenging problem. As study samples, human PDL stem cell sheets ready with recombinant human bone morphogenetic protein-2 (rhbmp-2) were positioned on micro/macro-porous biphasic calcium phosphate (MBCP) simulated dental roots [255]. In particular, researchers focused on how collagen fibers originate in cementum and how they adhere to the surface of roots. The results indicated that the material successfully promoted the regeneration of mineralized layer, withal, the collagen fibers inserted vertically or obliquely into the surface of MBCP.

Unfortunately, only using biomimetic cementum cannot restore periodontal function ideally. As mentioned above, periodontal tissue includes cementum, alveolar bone and PDL, which is a layered tissue organ with both soft and hard tissues. The soft tissue PDLs are arranged in a certain way to connect cementum and alveolar bone to provide stability for teeth in company with facilitate oral function [256]. The difficulty of periodontal tissue regeneration lies in the complex structural regeneration [257]. The current scientific tendency is to tackle alveolar bone, cementum, and cementum as a unit [236], then realize the tissue restoration according to the characteristics of each part.

Endogenous periodontal tissue regeneration can be achieved by simulating periodontal structure. The biomimetic of the natural periodontal hard/soft tissue interface was realized by constructing a hierarchical bilayer architecture [258]. Among the architecture, the PDL-like structure material is a parallel and ordered fiber structure made of a new generation of platelet concentrated growth factor (CGF) by microstamping technique. And the intrafibrillarly mineralized collagen (IMC) scaffold mimics alveolar bone-like structure made by self-assembly technology, showing bone-like transverse grain nanostructure and uniform porous microstructure. Ultimately, CGF arrays and IMC layers were mechanically combined to form a periodontal ordered CGF/IMC bilayer scaffold. The Young's modulus of the two layers of the scaffold were close to that of the natural PDL and alveolar bone, respectively. The bilayer scaffold succeeds in inducing stem cells to differentiate into the corresponding soft and hard tissues, while also activating the TGF-β1/Smad3 pathway to effectively repair the periodontal tissue.

Functional periodontal regeneration can be achieved by imitating stem cell regeneration microenvironment. Given the potential of endogenous ECM stem cell therapies in periodontal regeneration, mineralized ECM/dental pulp stem cell microspheres (MMCMs) were developed to simulate natural mesenchymal microsphere precursors [259]. The application of the biomineralization approach enhances the activity of the cells in the microspheres, in addition to the ability of osteogenesis and ECM secretion, which promotes periodontal defect healing by recruiting host stem cells. It is anticipated that by improving MMCMs, the inflammatory condition of periodontal tissue regeneration would be advanced, allowing clinical application of the material to get started as soon as possible.

Via integrating the aforementioned concepts, satisfactory periodontal regeneration can be achieved by replicating both the periodontal tissue structure and the biochemical microenvironment. 3D-bioprinting technology has demonstrated encouraging results in imitating the content and configuration of native periodontal tissue [260]. In this case, the decellularized extracellular matrix (dECM) from porcine dental follicle was added to methacrylate gelatin (GelMA) as bio-ink, then the PDL module and alveolar bone module were printed to create a biomimetic plus functional periodontal module. The addition of dECM raises the mechanical characteristics and biological activity of bio-ink while mimicking the biochemical microenvironment, triggering cell differentiation along with regulating immune system.

## Biomimetic bone substitute materials

Maxillofacial bone tissue plays an important physiological role in supporting, protecting and maintaining mineral stability [6, 261, 262]. The bone defects caused by trauma, tumor and genetic factors seriously affect the appearance, chewing, pronunciation and other physiological functions of patients [263–265]. Most minor bone tissue injuries and small bone defects can be healed by themselves, while the serious ones do not have such self-healing ability, which need grafts to fill the gaps and achieve the regeneration of the bone tissue [265, 266]. There are clinical methods for repairing severe bone abnormalities such as autogenous bone graft, bone allograft and xenograft bone graft [267–269]. However, these approaches have certain limitations. Disadvantages of autologous bone graft include high absorption rate, donor site disease and limited available bone. Bone allografts could lead to infection and induce immune rejection [15]. While xenogeneic bone graft may transmit pathogens from animals and induce immune rejection [5]. These approaches’ limiting properties led to the rise of studies in bone substitute materials. However, conventional bone substitute materials, such as metal bone repair materials, ceramic materials and polymers still have some shortcomings. Their biological activity, mechanical properties, osteogenic properties and other aspects still need to be improved [270, 271]. Therefore, the development of biomimetic bone substitute materials with better properties has become a hot trend in recent years. At present, there are two highly active research directions of biomimetic bone substitute materials, which refer to cell-free and cell-based bone regeneration materials [272]. The former is the biomineralization-inspired biomimetic material, while the latter is the biomimetic material based on bone tissue engineering.

### Biomineralization-inspired biomimetic materials

Bone is consisted of calcium phosphate crystals, cells, collagen and other large molecules [273]. Bone mineralization is similar to the dentin mineralization process mentioned above. It refers to the orderly accumulation of mineral crystals onto the organic ECM [274–276]. The main components of ECM involved in mineralization are type I collagen and non-collagen components. Type I collagen forms collagen fibers through self-assembly [277]. And the main role of it is to act as a template for mineralization of inorganic crystals. While non-collagen components are composed of NCP and some phospholipids, which act as signaling molecules during mineralization to induce mineral deposition [278]. Comparable to dentin mineralization, it is more significant to achieve intrafibrillar mineralization than extrafibrillar mineralization. The biomimetic inspiration of biomineralization-inspired biomimetic materials also comes from these two important components that play a key role in the mineralization process, including collagen fibers and non-collagen components. By imitating the connection between them, the researchers constructed intrafibrillarly mineralized scaffolds in vitro, mineralized them, and then implanted them in vivo for bone repair.

Next, we will focus on the significant biomimetic mineralized scaffold materials. First, the mineralized collagen scaffold contains the most similarities with bone in terms of composition. And NCP analogues have been used to construct fibrous mineralized collagen scaffolds recently. For example, some scholars have created hierarchical intrafibrillarly mineralized collagen (HIMC) by precisely adding a certain amount of PAA, and it has been proved that HIMC scaffolds can promote cell differentiation through in vitro cell experiments [279, 280]. However, the manufacturing of the HIMC stent does take a lengthy time, which is a drawback. Besides, the chitosan and the dopamine hydrochloride were used to construct a multi-layer scaffold structure. Meanwhile, the experiments showed that the scaffold has good biocompatibility and stability [281]. In addition, several metals in natural bone can also be combined with collagen scaffolds to stimulate bone tissue growth [282]. What’s more, silica can also penetrate into collagen fibers and silicify them [283, 284]. Some studies have shown that the strength of silicified collagen fibers was significantly higher than that of non-silicified ones [285]. Thus, it is believed that the development direction of biomimetic mineralization scaffolders in the future may be the intrafibrillar mineralization of a variety of mineral mixtures [286]. Recently, polymers were designed to create non-collagen scaffolds in place of collagen. The polymer-induced mineralization is stable, the biocompatibility is excellent, and the toughness is superior to collagen scaffolds [287].

### Biomimetic material used in bone tissue engineering

The three elements of bone tissue engineering include cells, growth factors and scaffold materials [288]. The scaffold is a reticular structure that supports and promotes bone cells’ adhesion as well as growth [289]. Many scholars have injected bioactive substances and stem cells into the scaffold material to simulate the microenvironment in vivo, and then stimulate the mineralization and repair of bone tissue [290]. This method can greatly avoid immune rejection from foreign implants. The ideal scaffold material should have the characteristics of good biocompatibility, absorbability, non-toxicity and sufficient mechanical strength [291]. However, increasing osteogenesis alone cannot satisfy clinical requirements, thus researches on functional scaffolds with vascularization and neuralization functions has attracted attention in recent years [292, 293]. The situ vascularized bone regeneration has been realized by 3D printing porous scaffolds and adding vascular endothelial cells [294, 295]. Besides, it was shown that magnesium ions and silicon ions have a certain role in the regulation and promotion of angiogenesis [296]. Based on this, silicon and magnesium ions were added to the scaffolds to promote angiogenesis, while zinc ions could also be employed for stimulating osteogenic differentiation [297, 298]. What’s more, some scholars have simulated natural bone piezoelectric properties to develop a multi-functional biomimetic piezoelectric scaffold with magnesium release ability [299]. Recent research showed that a biomimetic Ti-Mg composite was fabricated by pressureless infiltration of pure Mg melt into 3D printed Ti scaffold. The Mg-Ti composite possessed higher strengths than ascast pure Mg, and exhibited a lower Young’s modulus than dense Ti which further decrease adaptively during the degradation process of Mg to alleviate the stress shielding effect [300].

## Conclusions and future perspectives

Biomimetic materials have attracted much attention in the territory of oral medicine for its ability to mimic natural tissues. This review recapitulates recent developments and related applications of biomimetic oral materials. Nevertheless, no biomimetic oral materials are available on the market and the challenges of biomimetic materials research should not be ignored. More efforts should be devoted into biomimetic mineralization, biomimetic artificial restoration materials, tooth regeneration, periodontal and bone regeneration technologies to create the most cutting-edge and successful biological substitutes for various oral tissues.

In terms of biomimetic mineralization, the mineralization mechanism of tooth hard tissue is still controversial [301–303]. Thus, seeking the best constituent to construct the microenvironment will be hotspots in the future. The thickness of remineralized enamel is currently limited to micrometers, which is difficult to fabricate the overall structure of enamel. Concerning the biomimetic artificial restoration materials, even though they show excellent performance in mechanical properties, the materials manufactured by various methods have their own drawbacks, such as technical precision insufficiency. Tooth regeneration is recognized as the ideal option for treating tooth loss, but theories on how teeth form are still being investigated [304–306]. Techniques for achieving tooth regeneration (whole-tooth engineering and root regeneration) have not performed well in animal trials. In whole-tooth engineering, it is difficult to manage the location, shape, and capacity of the regenerated teeth. Meanwhile, the scaffold materials employed to construct the bio-root provoke immune reactions. Both of the abovementioned studies on tooth regeneration lack in vivo models and are far from clinical translation. In addition, regenerative techniques for the complex structure of periodontal tissue and the development of bone biomimetic materials are not yet mature.

Amid the development of manufacturing technologies of oral materials as well as the research progress of soft and hard tissue regeneration, the further improvement of biomimetic materials will become one of the significant fields in oral medicine. For one thing, it is essential to learn more about the development of relevant tissues, which is beneficial for choosing biomimetic mineralization materials and scaffold materials for regeneration engineering. For another, the consolidation of dependable manufacturing technologies may enable the production of biomimetic artificial restoration materials that are healthful, safe, and long-lasting. Finally, the implementation of animal experiments is required to update the benefits and drawbacks of biomimetic materials in order to help bring the materials to market successfully [307].

Biomimetic materials will undoubtedly develop into a new research trend and the standard method of treating oral diseases. Further developments of biomimetic materials are expected to give a prominent contribution to other fields [308]. By applying additional modeling and simulation techniques into the investigation of biomimetic oral materials, the best strategy to fabricate biomimetic components with superior performance is anticipated to be derived.

## Data Availability

The data presented in this study are available in articles.

## Abbreviations

ACP: Amorphous calcium phosphate
ADPs: Amelogenin-derived peptides
AEFC: Acellular extrinsic fiber cementum
AIP: Amorphous intergranular phase
AM: Additive manufacturing
aMBG: Aminated mesoporous bioactive glass
AMEL: Amelogenin
AMP: Antimicrobial peptides
bSi: Black silicon
CA: Contact angle
CaP: Calcium phosphate
CGF: Concentrated growth factor
CMCS: Carboxymethyl chitosan
CPICs: Calcium phosphate ion clusters
CPP: Casein phosphate polypeptide
CS: Chitosan
dECM: Decellularized extracellular matrix
DLP: Digital light processing
DPP: Dentin phosphoprotein
DSP: Dentin sialoprotein
DSS: Aspartate-serine-serine
ECM: Extracellular matrix
EMPs: Enamel matrix proteins
FHA: Fluoridated hydroxyapatite
GCHT: Glue-chrondrotin-hyaluronan-tri-copolymer
GelMA: Methacrylate gelatin
GTR: Guided tissue regeneration
HA: Hydroxyapatite
HIMC: Hierarchical intrafibrillarly mineralized collagen
hTDM: Human treated dentin matrix
ID-8: Ile-Asp-Ile-Asp-Ile-Asp-Ile-Asp
IMC: Intrafibrillarly mineralized collagen
LBL: Layer-by-layer
LRAP: Leucine-rich amelogenin peptide
MBCP: Micro/macro-porous biphasic calcium phosphate
MDG1: Mineral directing gelator
MMCMs: Mineralized ECM/ dental pulp stem cell microspheres
MMP: Matrix metalloproteinases
MPP3: PGEKADRAEKADRA
NAA: Non-amelogenin analog
NACP: Nanoparticles of amorphous calcium phosphate
NCP: Non-collagenous protein
nFHA: Fluorine-containing nano-hydroxyapatite
nHA: Hydroxyapatite nanoparticles
NPs: Nanopillars
NRDs: Nanorods
P11-4: CH3CO-Gln-Gln-Arg-Phe-Glu-Trp-Glu-Phe-Glu-Gln-Gln-NH2
PA: Peptide-amphiphile
PAA: Polyacrylic acid
PAAm: Polyallylamine
PAMAM: Polyamidoamine
PASP: Poly aspartic acid
PDA: Polydopamine
PDLs: Periodontal ligaments
PDMS: Polydimethylsiloxane
PEG: Polyethylene glycol
PET: Polyethylene terephthalate
PGA/PLLA: Polyglycolate/poly-L-lactate
PILP: Polymer-induced liquid-precursor
PLGA: Poly-L-lactate-co-glycolate
PMMA: Polymethyl methacrylate
PS-block-PMMA: Polystyrene-block-polymethy lmethacrylate
pTDM: Porcine TDM
PVA: Polyvinyl alcohol
PVPA: Polyvinylphosphonic acid
RGD: Arginine-glycine-aspartate
rhbmp-2: Recombinant human bone morphogenetic protein-2
rm180: The (180 amino acid-long) amelogen
shADP5: Shortened 22-amino-acid long amelogenin-derived peptide
SSD: Ser-ser-asp
TA: Tannic acid
TCP: Tricalcium phosphate
TEA: Triethylamine
TESPSA: Triethoxysilylpropyl succinic anhydride
TPMS: Triply periodic minimal surface
VFOM: Viscoelastic figures of merit
Y-TZP: Yttria-stabilized tetragonal zirconia polycrystalline
ZnO-PSBMA: ZnO-poly N-isopropylacrylamide
